# Can clinical prediction models assess antibiotic need in childhood pneumonia? A validation study in paediatric emergency care

**DOI:** 10.1371/journal.pone.0217570

**Published:** 2019-06-13

**Authors:** Josephine van de Maat, Daan Nieboer, Matthew Thompson, Monica Lakhanpaul, Henriette Moll, Rianne Oostenbrink

**Affiliations:** 1 Department of General Paediatrics, Erasmus MC–Sophia Children’s Hospital, Rotterdam, The Netherlands; 2 Department of Public Health, Erasmus MC, Rotterdam, The Netherlands; 3 University of Washington, Department of Family Medicine, Seattle, United States of America; 4 Population, Policy, Practice Program, UCL Great Ormond Street Institute of Child Health, London, United Kingdom; Fundacao Oswaldo Cruz, BRAZIL

## Abstract

**Objectives:**

Pneumonia is the most common bacterial infection in children at the emergency department (ED). Clinical prediction models for childhood pneumonia have been developed (using chest x-ray as their reference standard), but without implementation in clinical practice. Given current insights in the diagnostic limitations of chest x-ray, this study aims to validate these prediction models for a clinical diagnosis of pneumonia, and to explore their potential to guide decisions on antibiotic treatment at the ED.

**Methods:**

We systematically identified clinical prediction models for childhood pneumonia and assessed their quality. We evaluated the validity of these models in two populations, using a clinical reference standard (1. definite/probable bacterial, 2. bacterial syndrome, 3. unknown bacterial/viral, 4. viral syndrome, 5. definite/probable viral), measuring performance by the ordinal c-statistic (ORC). Validation populations included prospectively collected data of children aged 1 month to 5 years attending the ED of Rotterdam (2012–2013) or Coventry (2005–2006) with fever and cough or dyspnoea.

**Results:**

We identified eight prediction models and could evaluate the validity of seven, with original good performance. In the Dutch population 22/248 (9%) had a bacterial infection, in Coventry 53/301 (17%), antibiotic prescription was 21% and 35% respectively. Three models predicted a higher risk in children with bacterial infections than in those with viral disease (ORC ≥0.55) and could identify children at low risk of bacterial infection.

**Conclusions:**

Three clinical prediction models for childhood pneumonia could discriminate fairly well between a clinical reference standard of bacterial versus viral infection. However, they all require the measurement of biomarkers, raising questions on the exact target population when implementing these models in clinical practice. Moreover, choosing optimal thresholds to guide antibiotic prescription is challenging and requires careful consideration of potential harms and benefits.

## Introduction

Community-acquired pneumonia is the second largest cause of childhood mortality worldwide [1]. Despite improvements over the past decades, lower respiratory tract infections are still responsible for 103.3 deaths per 100,000 people in children under five years globally, with large differences across regions [2]. Respiratory tract infections are also a common reason for emergency department (ED) visit and the most frequent indication for antibiotic prescription in children [1, 3]. Discriminating bacterial infections that require antibiotic treatment from viral, self-limiting disease is one of the biggest diagnostic challenges in childhood pneumonia. Chest x-ray is no longer recommended as the gold standard for bacterial pneumonia [4], and routinely available biomarkers are not pathognomonic for this diagnosis [5]. At the same time, accurate diagnosis of bacterial infection is crucial, since misuse of antibiotics is associated with increased antimicrobial resistance, which in turn also causes morbidity and mortality [6]. Current antibiotic prescription for suspected pneumonia in Western countries ranges from 23–59% with wide acknowledgement that a considerable proportion of these antibiotics are not necessary [3, 7].

In order to standardize the evaluation and treatment of children suspected of pneumonia, clinical decision support systems could be useful tools to classify children into a high or low risk profile [8]. Multiple clinical prediction models for childhood pneumonia have been developed. Even though their current use in clinical practice is limited, they may play a role as treatment decision support, thereby improving rational antibiotic prescription. However, since those models are mainly developed with chest x-ray as their reference standard, it is unclear if they can also validly predict a clinically based diagnosis of pneumonia. Moreover, the question is whether these models can be translated into clinical practice by guiding decisions on antibiotic treatment.

This study aims to systematically search available clinical prediction models for childhood pneumonia in ED settings in high-income countries, to evaluate their validity using a new, clinical diagnosis reference standard, and to explore their potential to guide decisions on antibiotic treatment.

## Methods

### Selection and quality assessment of prediction models

A systematic search for prediction models of childhood pneumonia was performed in Embase, Medline Ovid, Web of science, PubMed and Google scholar in September 2017. We included studies on diagnosis and treatment of uncomplicated childhood pneumonia in ED settings in Western countries published since 2000 (see search strategy and exclusion criteria, S1 Text). JvdM and BK performed the selection independently, discrepancies were discussed within the research group and decided using consensus.

We evaluated the clinical prediction models for their quality and diagnostic value. Quality assessment was performed by JvdM and checked by RO, using the QUADAS-2 tool for diagnostic studies [9]. We assessed their level of validation using the guideline proposed by the Evidence-Based Working Group [10] with one added category as described by Reilly [11], ranging from level 1 ‘derivation of the model without validation’ to level 5 ‘proven by broad impact analysis’.

### Validation study

#### Validation populations

We retrospectively evaluated the validity of the identified prediction models in two study populations [12, 13]. Population 1 included 248 children aged 1 month to 5 years presenting at the ED in 2012–2013 with fever and cough or dyspnoea, from a prospective study at the Erasmus MC—Sophia, Rotterdam, the Netherlands [12]. Population 2 included 301 children aged 3 months to 5 years presenting with fever and respiratory symptoms at a paediatric assessment unit at the University Hospitals Coventry and Warwickshire NHS Trust, United Kingdom (UK), in 2005–2006 [13]. In both databases children with comorbidity related to increased risk of bacterial infection or complications were excluded, such as severe neurological impairment, immunodeficiency and severe pulmonary or cardiac defects. Follow-up was available for both populations, reducing the risk of missing (untreated) serious infections. The studies in these populations were approved by the Medical Ethics Committee of the Erasmus MC (Rotterdam) and the Coventry Local Research Ethics Committee. Written informed consent was obtained for both populations [12, 13].

#### Reference standard

As chest x-ray is no longer recommended as a gold standard in clinical practice, the diagnosis of bacterial pneumonia is mostly a clinically based diagnosis. A model that may reflect this clinical approach, is an algorithm published by Herberg et.al., classifying the potential aetiology of febrile illness in children [14]. For this study, we used a reference standard adapted to this model, classifying patients’ cause of respiratory tract infection from bacterial to viral (see S1 Fig). First, we pre-specified what working diagnosis would be classified as ‘bacterial syndrome’, ‘viral syndrome’ or ‘unknown bacterial/viral’, the first step of the algorithm. Then we categorized all patients based on their working diagnosis as documented in the different databases. We used the working diagnosis that was attributed by the attending clinician at the end of the ED visit, based on patient assessment and routine diagnostic tests. As a second step, we used identification of bacteria or viruses and CRP-level (>60 mg/l or ≤60mg/l) to further differentiate the clinical diagnosis. Diagnostic tests from routine care included viral PCR of nasopharyngeal swab and blood cultures, as performed at the discretion of the clinician. Given a low number of pathogens identified we had few definite diagnoses, so we classified patients into to five categories: definite or probable bacterial (1), bacterial syndrome (2), unknown bacterial or viral (3), viral syndrome (4) and definite or probable viral (5). For example, a child presenting with bronchiolitis (viral syndrome at first step), no virus or bacteria identified and a CRP-level of >60mg/l would be classified as having a viral syndrome. A child with a working diagnosis of pneumonia (unknown viral/bacterial at first step), the CRP-level would lead to either bacterial syndrome (in case of high CRP), viral syndrome (in case of low CRP) or remain unknown bacterial/viral (in case of no CRP performed). Patients with a bacterial and viral co-infection were classified as bacterial infection, given the consequences for treatment.

#### Statistical analysis

Missing values were imputed 10 times using the mice package in R (version 3.3.2), resulting in 10 separate datasets with complete (imputed) information. The imputation model included information about clinical signs and symptoms, referral, diagnostic tests and treatment. We performed all analyses of the validation on the 10 imputed datasets and then averaged the results [15]. When a variable of a prediction model was completely missing in our database, multiple imputation was not possible and we used a proxy (e.g. ‘retractions’ as a proxy variable for ‘dyspnoea’, if ‘dyspnoea’ was not available). For continuous variables, the prevalence of that variable in the original derivation population of the prediction model was used (mean imputation) [16]. CRP-level was truncated at the level of 225 mg/L, following the study of Nijman [17].

We evaluated the validity of those prediction models of which more than 50% of the predictors were available in our database, assuming this as a minimum for credible predictions [16]. We calculated the risk of bacterial pneumonia using each of the included prediction models for all children in our study populations, illustrated by histograms and boxplots. To measure performance, we calculated the ordinal c-statistic (ORC)–a measure similar to the area under the receiver-operating-curve (AUC), but for ordinal instead of dichotomous outcomes. This statistic can be interpreted as the probability that two cases of randomly selected outcome categories are correctly ranked [18]. We defined models with an ORC of at least 0.55 as performing well and explored their potential to guide antibiotic prescription. For this purpose, we evaluated the harms and benefits of withholding antibiotics in low-risk patients, compared to the observed usual care in which treatment decisions were based on clinical judgment and routine diagnostic tests. Benefit was defined as the potential reduction of antibiotic prescription and harm as the potential risk of under treatment. Under treatment was defined as children that were classified as having a bacterial infection and who had been treated with antibiotics, but whom the prediction model classified as low-risk. We explored different thresholds for the prediction models to define low-risk and evaluated their effect on harms and benefits. All analyses were performed using SPSS (IBM version 24.0) and R (version 3.3.2).

## Results

### Identification, quality and original performance of prediction models

We identified 4324 unique articles (after removal of duplicates). Based on title and abstract 4176 articles were excluded as not relevant (see S2 Fig). After full-text selection and searching references, 11 articles were eligible for inclusion (see Table 1). Eight were primary derivation studies, describing different prediction models [17, 19–25], three were validation or impact studies of three of these models [12, 26, 27] and one derivation study also included the validation of another model [25]. Even though VandenBruel’s model was derived mainly in general practice setting, it was also validated in an ED setting, and therefore included in our study. Most studies included children up to the age of 16, but the majority of the included patients in all studies were under five. Most studies had radiographic pneumonia as their reference standard, except for VandenBruel’s study that used hospitalization for radiographic pneumonia as its reference standard (Table 1). All prediction models aimed to improve clinical decision-making in the child suspected of bacterial pneumonia. Three studies mainly focused on decisions on diagnostic tests [19, 21, 23]; the other studies also mentioned the potential of the models to improve management decisions on antibiotic treatment, admission or referral [17, 20, 22, 24, 25].

**Table 1:** Characteristics of clinical prediction models.

Clinical prediction rule	Setting	Population	Original reference standard	Prevalence pneumonia	Statistical model	Predictor variables	Performance				Level of evidence**	QUADAS-2
*Risk classification (high versus low risk)*							*Sensitivity*	*Specificity*	*LR+*	*LR-*		*risk of bias / concern applicability*
**1. Mahabee (2005)[23]**	US	2m - 5y, cough + 1 of following: labored/ rapid/noisy breathing; chest/abdominal pain; fever	radiographic pneumonia	44/510 (8.6)	MLRM	age≥12 months, respiratory rate ≥50/min, oxygen saturation ≤96%, nasal flaring in age <12months	63.6	77	2.8	0.5	1	low / low
**2. Bruel, van den (2007)[20]**	BE*	< 17y, acute illness	hospital admission for radiographic pneumonia	15/3981 (0.4)	CART	dyspnea, 'something is wrong'	93.8	93.2	13.9	0.07	3	low / high
Verbakel (validation 1, 2013)[26]	NL	"	"	17/506 (3.3)			94.1	44.6	1.7	0.13		NA, different datasets
Verbakel (validation 2, 2013)	UK	"	"	131/2687 (4.9)			92.4	41.4	1.58	0.18		
Verbakel (validation 3, 2013)	NL	"	"	114/1750 (6.5)			65.8	43.1	1.16	0.79		
Verbakel (validation 4, 2013)	NL	"	"	54/595 (9.1)			81.5	45.5	1.49	0.41		
Verbakel (validation 5, 2013)	UK	"	"	67/700 (9.6)			26.9	89.1	2.46	0.82		
**3. Neuman (2011)[21]**	US	< 21, chest X-ray for suspected pneumonia	radiographic pneumonia	422/2574 (16.4)	CART	oxygen saturation ≤92%, history of fever, wheezing, focal rales, chest pain, focal decreased breath sounds	90.1	21.6	1.2	0.4	1	some / low
*Probability (predicted risk in %)*							*AUC*					
**4. Lynch (2004)[19]**	US	1-16y, chest X-ray for suspected pneumonia	radiographic pneumonia	204/570 (35.8)	MLRM	fever, decreased breath sounds, crackles, tachypnea	0.67				3	some / low
Bilkis (validation, 2010)[27]	US	"		179/257 (69.6)			0.7					some / some
**5. Oostenbrink (2013)[24]**	NL	1m - 16y, fever and cough	nodular infiltration or consolidation on radiograph / rule out pneumonia by noneventful followup / consensus	78/504 (15.5)	MLRM	ill appearance, tachypnea, O2 <94%, CRP	0.79				3	some / low
Oostenbrink (validation 1, 2013)	NL	"		58/420 (13.8)			0.81					
Oostenbrink (validation 2, 2013)	NL	"		27/366 (7.4)			0.86					
**6. Craig (2010)[22]**	AU	<5y, fever	consolidation on radiograph	533/15781 (3.4)	MLRM	general appearance, cough, temperature, breathing difficulty, abnormal chest sounds, chronic disease, capillary refill time, urinary symptoms, elevated respiratory rate, crackles, pneumococcal vaccine status, elevated heart rate, felt hot, meningococcal vaccine state, infectious contacts, crying, fluid intake, respiratory symptoms, diarrhoea, bulging fontanelle, male sex, focal bacterial infection, abnormal ear/nose/throat signs, age, rash, stridor, wheeze	0.84				2	low / low
Craig (validation, 2010)	AU	"		193/5584 (3.5)			0.84					low / low
**7. Nijman (2013)[17]**	NL	1m - 15y, fever	nodular infiltration or consolidation on radiograph; rule out pneumonia by noneventful followup	171/2717 (6.3)	MLRM	age, sex, duration of fever, temperature, respiratory rate, heart rate, oxygen saturation, capillary refill, retractions, ill appearance, CRP	0.81				4	low / low
Nijman (validation, 2013)	NL	"		59/487 (12.1)			0.81					low / low
De Vos (validation, 2015) [12]	NL	"		33/439 (7.5)			0.83					low / low
**8. Irwin (2017)[25]**	US	<16y, (history of) fever	respiratory symptoms, signs and focal consolidation on radiograph	63/532 (12)	MLRM	CRP, respiratory rate, normal air entry, resistine, procalcitonin	0.84				1	low / low

m = months, y = years, ED = emergency department, GP = general practice, US = United States of America, BE = Belgium, NL = the Netherlands, AU = Australia, UK = United Kingdom

CART = classification and regression tree, MLRM = multivariable linear regression model, LR+ = positive likelihood ratio, LR- = negative likelihood ratio, AUC = area under the receiver operating curve

^a^derived in general practice and emergency department, validated in ED

^b^as described by Reilly (range 1 (only derived) to 5 (proven by broad impact analysis)[11]

In general the quality of the prediction models was moderate (see Table 1 and S3 Fig) with 3 models having some risk of bias [19, 21, 24] and one study with concerns about the applicability [20]. Nijman’s model was evaluated most thoroughly including impact analysis [17]. The models by VandenBruel, Lynch and Oostenbrink were broadly validated in previous studies [19, 20, 24]; those by Mahabee-Gittens, Neuman, Craig and Irwin were only derived or validated in one setting by the original authors [21–23, 25].

Three prediction models provided a risk classification (high versus low risk), based on the presence of specific symptoms [20, 21, 23]. Of these models, sensitivity at model development was moderate to good, with varying specificity (see Table 1). Only VandenBruel’s model was validated in different settings, performing poorly due to high sensitivity and low specificity in three settings, the opposite in another setting, and in a last setting both poor sensitivity and specificity [26]. The other four prediction models provided a probability (predicted risk in %) of pneumonia, based on a multiple logistic regression model [17, 19, 24, 25]. These models showed moderate to good performance at development (AUC ranging from 0.67 to 0.84) as well as in the validation studies [22, 24, 26].

### Validation study

Table 2 shows the baseline characteristics of the two populations. Using the clinical diagnosis, bacterial infection rate ranged from 9–17% and 38–41% were classified as ‘unknown’. Of this latter category 74–87% recovered without antibiotics. We included seven prediction models in our validation study. We did not assess validity of Craig’s model as only 14/28 variables were present in both databases. Lynch–having only 2/4 variables available–was not validated in the Coventry database. The supplementary S1 Table gives an overview of all variables and proxies of the validated prediction models. Mahabee-Gittens published a regression model providing a probability, but the coefficients to calculate this probability were not available from the author [23]. We therefore used the presence of one or more of the included variables classifying patients at high risk of bacterial pneumonia. VandenBruel published a general prediction model for febrile children, and one for pneumonia; for this review we only used the pneumonia model [20]. Neuman used a decision tree to classify patients into 3 categories (high/intermediate/low risk of pneumonia) [21]. In this model ‘history of fever’ discriminated intermediate from low risk, but since fever was an inclusion criteria of all our validation populations, only high and low risk patients were identified, based on the first step of the decision tree (oxygen saturation <92%).

**Table 2:** Baseline characteristics of validation populations.

	Rotterdam, n = 248	Coventry, n = 301
*Predictor variables*	*median (IQR) or n(%)*	*median (IQR) or n(%)*
Age (months)	14 (7–27)	19 (12–31)
Gender (male)	148/248 (60%)	174/301 (58%)
Temperature (C°)	38.2 (37.4–39.1)	38.2 (37.5–39.1)
Duration of fever (days)	3 (2–4)	not available
Tachypnea	81/183 (44%)	154/258 (60%)
Tachycardia	66/207 (32%)	191/294 (65%)
Oxygen saturation (%)	98 (97–100)	97 (95–98)
Ill appearance	35/149 (23%)	1/301 (0%)
Dyspnoea	106/248 (43%)	81/301 (27%)
Decreased breath sounds	12/136 (9%)	not available
Crackles	30/127 (24%)	not available
Focal rales	67/151 (44%)	not available
Retractions	68/107 (64%)	not available
Nasal flaring	29/58 (50%)	not available
Prolonged capillary refill (>2sec)	10/53 (19%)	58/187 (31%)
*Diagnostics and treatment*	* *	* *
CRP measured	94/248 (38%)	109/301 (36%)
CRP (mg/L)	16 (7–42)	45 (19–122)
X-ray performed	42/248 (17%)	67/301 (22%)
Antibiotics prescribed	51/248 (21%)	105/301 (35%)
*Clinical diagnosis (S1 Fig)*		* *
Definite or probable bacterial	18/248 (7%)	37/301 (12%)
Bacterial syndrome	4/248 (2%)	16/301 (5%)
Unknown	94/248 (38%)	122/301 (41%)
Viral syndrome	59/248 (24%)	72/301 (24%)
Definite or probable viral	73/248 (29%)	54/301 (18%)

IQR = interquartile range

#### Performance of prediction models

**Fig 1:**
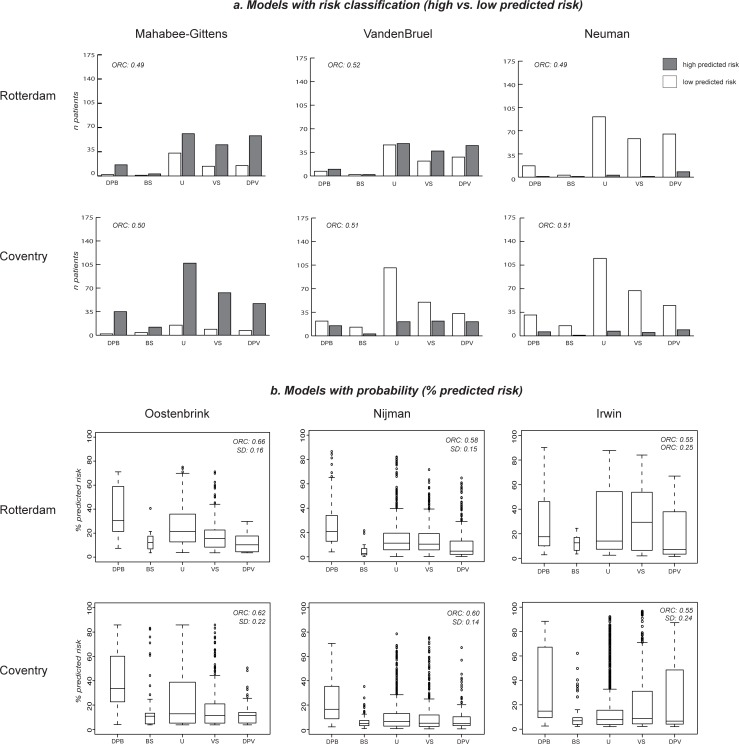
Performance of prediction models. **a. Models with risk classification (high vs. low predicted risk) b. Models with probability (% predicted risk).** DPB = definite or probable bacterial, BS = bacterial syndrome, U = unknown, VS = viral syndrome, DPV = definite or probable viral; ORC = ordinal c-statistic; SD = standard deviation.

The performance of the three models with a risk classification (high/low risk) is shown in Fig 1A. The white bars indicate the number of children with predicted low risk of pneumonia and the grey bars the number of patients with predicted high risk, across the five reference standard categories (bacterial to viral infection). For example, when we used Mahabee-Gittens’ model to predict the risk of having a bacterial pneumonia in our two validation populations, we observed that this model predicts most children as having a high risk of pneumonia (grey bars), including most children with viral infections. Using VandenBruel’s model, we observed low as well as high predicted risks across all 5 diagnosis categories. Almost all children were assigned to a low risk group using Neuman’s model, including children with bacterial infections.

Fig 1B shows the performance of the prediction models providing a probability. Again, predictions are shown across the five diagnosis categories for each model and for both populations, illustrated by a boxplot. Lynch’s model predicted high risk of pneumonia (around 90%) for all children, with little variation across the different outcome categories (see S4 Fig), and did not contribute to discrimination between bacterial or viral disease. The models by Oostenbrink, Nijman and Irwin assigned higher risks to children with bacterial infections than to the children with viral infections, confirmed by a moderate ordinal c-statistic of ≥0.55 (see Fig 1B).

To assess the clinical relevance of these findings, we explored the potential of the last three models to define low-risk patients possibly not needing antibiotic treatment. For example, applying a risk threshold of 10% using Nijman’s model would classify 130 children (52%) in the Rotterdam population as being at low risk of bacterial pneumonia (see Table 3, details in S2 Table). Of these children 16 were currently treated with antibiotics. If this threshold would be used in clinical practice, and antibiotics would be withheld in all low-risk children, the overall antibiotic prescription rate would reduce from 21% (observed antibiotic prescription) to 14% (expected antibiotic prescription) in the Rotterdam population and from 35% to 16% in the Coventry population (Table 3). The potential risk of under treatment (e.g. withholding antibiotics in children with a bacterial infection who were currently treated with antibiotics) would be 2% (Rotterdam) and 5% (Coventry). Similar benefits and harms were observed when applying the models of Oostenbrink and Irwin. A threshold of 15% would lead to greater reduction in antibiotic prescription, but at a higher risk of under treatment.

**Table 3:** Clinical consequences of using prediction models to guide antibiotic prescription.

	Rotterdam, n = 248	Coventry, n = 301
**Observed antibiotic prescription, n (%)**	51 (21%)	105 (35%)
**Predictions by Nijman's model**		
***Threshold 10%***	*Rotterdam*	*Coventry*
Number of children below threshold **(low-risk group)**	130 (52%)	193 (64%)
Expected antibiotic prescription when guided by threshold **(benefit)**	35 (14%)	49 (16%)
Expected under treatment when prescription was guided by threshold **(harm)**^a^	5 (2%)	15 (5%)
***Threshold 15%***		
Number of children below threshold	167 (67%)	229 (76%)
Expected antibiotic prescription when guided by threshold	28 (11%)	36 (12%)
Expected under treatment when prescription was guided by threshold^a^	8 (3%)	22 (7%)
**Predictions by Oostenbrink's model**		
***Threshold 10%***	*Rotterdam*	*Coventry*
Number of children below threshold	69 (28%)	94 (31%)
Expected antibiotic prescription when guided by threshold	44 (18%)	77 (26%)
Expected under treatment when prescription was guided by threshold^a^	0 (0%)	8 (3%)
***Threshold 15%***		
Number of children below threshold	110 (44%)	178 (59%)
Expected antibiotic prescription when guided by threshold	35 (14%)	51 (17%)
Expected under treatment when prescription was guided by threshold^a^	2 (1%)	13 (4%)
**Predictions by Irwin's model**		
***Threshold 10%***	*Rotterdam*	*Coventry*
Number of children below threshold	100 (40%)	155 (51%)
Expected antibiotic prescription when guided by threshold	38 (15%)	64 (21%)
Expected under treatment when prescription was guided by threshold^a^	5 (2%)	15 (5%)
***Threshold 15%***		
Number of children below threshold	120 (48%)	198 (66%)
Expected antibiotic prescription when guided by threshold	33 (13%)	48 (16%)
Expected under treatment when prescription was guided by threshold^a^	8 (3%)	22 (7%)

^a^ Number of children with a bacterial infection who were treated with antibiotics, but who were classified as low-risk according to the used prediction model and threshold

## Discussion

We identified eight clinical prediction models for childhood pneumonia by literature review. Following changing perspectives on a relevant reference standard for childhood pneumonia, we could assess the validity of seven of them for a clinical diagnosis of bacterial, unknown bacterial/viral or viral infection. Three models–with good original performance and quality–assigned a higher risk to children with bacterial infection than to those with viral infection, with the potential of proper selection of children who may recover without antibiotics.

An important strength of our study is the broad validation of multiple prediction models in prospective cohorts including over 500 patients in two different European acute care settings. Our populations were rather heterogeneous in terms of their clinical characteristics, increasing the generalizability of our findings. A limitation is the heterogeneity of the information available, and missing values in general, which is related to the use of already existing datasets. We have accounted for this by multiple imputation or by using proxies where possible. Another limitation is the retrospective classification of the clinical diagnosis, based on the working diagnosis by the treating physician not blinded for clinical features and diagnostic tests. Because none of these clinical features or tests alone determined classification into a final diagnosis category, we believe this potential bias is limited. Diagnostic tests were performed at the discretion of the treating clinician, and included chest x-rays mainly. For 22 patients a definite viral or bacterial test was recorded to be positive, however, we had no data on the total performed viral/bacterial tests. Previous studies in these settings have shown that these are performed in about 10% of febrile children [12, 13]. Validity assessment of the model by Mahabee-Gittens was limited by the absence of the original coefficients. Of Irwin’s model only 3 out of 5 predictor variables were present, for the other two variables we used mean imputation. This may have underestimated the model’s discriminative value; but given the small effect sizes of the missing variables, we consider this effect limited [16].

We should appreciate several differences between our study populations and the populations the models were originally derived on. Since our populations included febrile children at the ED, it is not surprising that we observed less variability in the predicted probabilities in the validation of Neuman and Lynch’ models, since fever was one of their predictor variables. Furthermore, differences in pneumonia prevalence in the derivation populations (6–36%) of the models may explain systematic differences in predicted probabilities in 4 models [17, 19, 24, 25, 28]. In general, correcting for this involves recalibration (calibration-in-the-large) of the model to a new target population [28]. However, this type of recalibration does not influence discrimination (the ordinal c-statistic), and thus not our conclusions. It may, however, explain the variable impact the suggested thresholds have using the different models. Next, the type of reference standard (radiographic pneumonia vs. clinical diagnosis) differed between derivation and validation studies, as was the purpose of our study. Given the diagnostic limitations of chest X-rays, we chose to define our reference standard following Herberg’s classification [14]. It must be noted that this choice was not proposed as a new gold standard, but rather used as a model that may reflect our best current practice. In our aim to translate prediction models into clinical practice, we observed that the performance varied by type of model. We observed that the models using the probability scale had better diagnostic performance (reflected by a higher ORC statistic) than those using a risk classification (high/low risk). This can partly be explained by the ability to adjust risk thresholds–with a direct link to the harm-benefit ratio–more easily in models using the probability scale. Models using a risk classification have a fixed threshold and lack this flexibility and may therefore show lower diagnostic performance when validated according to a new reference standard.

In order to improve rational use of antibiotics in children with respiratory infections, there is a need to improve discrimination between bacterial and viral, self-limiting disease. We showed that three of seven tested clinical prediction models could identify a low-risk group of children with self-limiting disease in an ED population fairly well and we believe those three have the potential to improve treatment decisions. Those models include a combination of signs of general illness and/or respiratory distress and biomarkers. The availability of biomarkers will influence the feasibility of implementation of these models in clinical practice. The models of Oostenbrink and Nijman include CRP measurement, Irwin’s model includes CRP, procalcitonin and resistin. Given the wide availability of point-of-care CRP tests the first two models will be most feasible for routine use in the ED.

Another important challenge to be faced before prediction models can be implemented as decision tools in clinical practice is to choose optimal decision thresholds, adapted to the appropriate target population. A balance is needed between the benefit of reducing unnecessary antibiotic prescription and the harm of potential under treatment of bacterial infections. The prior risk of severe illness in a population is an important consideration. For example, in settings with high prevalence of comorbidity, the course of pneumonia will generally be more severe and missing a serious infection will have worse consequences than in a low-risk population. Next, the natural course of the disease should be taken into account. Last, access to (good quality) healthcare is important. In a setting with limited possibility for patient follow-up, potential risks of under treatment will higher. Given the natural course of pneumonia (developing over days instead of hours), a watchful waiting approach instead of immediate antibiotic treatment in children with uncomplicated pneumonia with a predicted risk <10–15% might be justified in settings with good access to care, in the presence of a proper safety-netting strategy for unexpected disease course. In low resource settings or high-risk populations lower thresholds may be reasonable. Before implementing treatment interventions based on these prediction models in clinical practice, a prospective study is needed to evaluate the overall impact of treating children according to such a prediction model, compared to usual care. Such a study should assess the feasibility and safety of the suggested thresholds for that specific setting.

Three out of seven clinical prediction models for pneumonia could discriminate fairly well between a new reference standard of bacterial and viral infection in children presenting at the ED. However, they all require the measurement of biomarkers, raising questions on the exact target population when implementing these models in clinical practice. Moreover, choosing optimal decision to guide antibiotic prescription is challenging and requires careful consideration of potential harms and benefits. Future research should focus on the feasibility and safety of treatment based on chosen decision thresholds for specific settings.

## Supporting information

S1 Text Search strategy and in/exclusion criteria for literature review. (PDF)

S1 Fig Classification of febrile illness. (PDF)

S2 Fig Flowchart of the selection process. (PDF)

S3 Fig QUADAS-2 quality assessment. (PDF)

S4 Fig Performance Model Lynch. (PDF)

S1 Table Missings and proxies of predictor variables. (PDF)

S2 Table Detailed classification of risk groups based on different prediction models. (PDF)
