# Supplementary material for: Can clinical prediction models assess antibiotic need in childhood pneumonia? A validation study in paediatric emergency care
Source: PLoS One. 2019 Jun 13;14(6):e0217570. doi: 10.1371/journal.pone.0217570 (PMC6563975; doi:10.1371/journal.pone.0217570)
Supplement: S1 Text — (PDF) [file pone.0217570.s001.pdf]

Supplementary Material 1. Search strategy and in/exclusion criteria for systematic review

Original search strategy, April 2016

Database	Number of articles	Number of articles after deleting duplicates
Embase.com	2676	2646
Medline Ovid	1466	310
Web of science	1462	610
PubMed publisher	52	31
Google scholar	223	158
**Total**	**5879**	**3755**

Embase.com 2676
('pneumonia'/exp OR (pneumoni* OR Bronchopneumoni* OR Pleuropneumoni*):ab,ti OR (('respiratory tract infection'/de OR 'lower respiratory tract infection'/de OR 'viral respiratory tract infection'/de OR (((respirator* NEAR/6 infection*) NOT (upper NOT lower))):ab,ti) AND ('antibiotic agent'/exp OR (antibiotic*):ab,ti))) AND ('practice guideline'/exp OR 'decision making'/exp OR 'decision support system'/exp OR 'decision tree'/exp OR ((guideline* OR ((decision OR prediction*) NEAR/3 (making OR support* OR tree* OR model* OR model* OR algorithm* OR triage* OR protocol* OR principle* OR aid OR aids))):ab,ti OR ((guideline* OR decision* OR model OR tree OR prediction* OR model* OR algorithm* OR triage* OR protocol* OR principle*) NEAR/6 (develop* OR propose* OR new OR novel OR validat* OR Evaluat* OR implement* OR modif*)):ab,ti)) AND (child/exp OR 'pediatrics'/de OR (preschool* OR child* OR schoolchild* OR infan* OR toddler* OR pediatric* OR paediatric*):ab,ti) AND [english]/lim NOT ([Conference Abstract]/lim OR [Letter]/lim OR [Note]/lim OR [Editorial]/lim)

Medline Ovid 1466
(exp "pneumonia"/ OR (pneumoni* OR Bronchopneumoni* OR Pleuropneumoni*).ab,ti. OR (("Respiratory Tract Infections"/ OR (((respirator* ADJ6 infection*) NOT (upper NOT lower))).ab,ti.) AND ("antibiotic agent"/ OR (antibiotic*).ab,ti.))) AND (exp "guideline"/ OR exp "Decision Making"/ OR "Decision Support Systems, Clinical"/ OR "Decision Support Techniques"/ OR "Decision Trees"/ OR ((guideline* OR ((decision OR prediction*) ADJ3 (making OR support* OR tree* OR model* OR model* OR algorithm* OR triage* OR protocol* OR principle* OR aid OR aids))).ab,ti. OR ((guideline* OR decision* OR model OR tree OR prediction* OR model* OR algorithm* OR triage* OR protocol* OR principle*) ADJ6 (develop* OR propose* OR new OR novel OR validat* OR Evaluat* OR implement* OR modif*)).ab,ti.)) AND (exp child/ OR infant/ OR "pediatrics"/ OR (preschool* OR child* OR schoolchild* OR infan* OR toddler* OR pediatric* OR paediatric*).ab,ti.) AND english.la. NOT (letter OR news OR comment OR editorial OR congresses OR abstracts).pt.

Web of science 1462
TS=(((pneumoni* OR Bronchopneumoni* OR Pleuropneumoni*) OR (((((respirator* NEAR/5 infection*) NOT (upper NOT lower)))) AND ((antibiotic*)))) AND (((guideline* OR ((decision OR prediction*) NEAR/2 (making OR support* OR tree* OR model* OR model* OR algorithm* OR triage* OR protocol* OR principle* OR aid OR aids))) OR ((guideline* OR decision* OR model OR tree OR prediction* OR model* OR algorithm* OR triage* OR protocol* OR principle*) NEAR/5 (develop* OR propose* OR new OR novel OR validat* OR Evaluat* OR implement* OR modif*)))) AND ((preschool* OR child* OR schoolchild* OR infan* OR toddler* OR pediatric* OR paediatric*))) AND LA=(english) AND DT=(article)

PubMed publisher 52
("pneumonia"[mh] OR (pneumoni*[tiab] OR Bronchopneumoni*[tiab] OR Pleuropneumoni*[tiab]) OR (("Respiratory Tract Infections"[mh] OR (((respirator*[tiab] AND infection*[tiab]) NOT (upper[tiab] NOT lower[tiab])))) AND ("antibiotic agent"[mh] OR (antibiotic*[tiab])))) AND ("guideline"[mh] OR "Decision Making"[mh] OR "Decision Support Systems, Clinical"[mh] OR "Decision Support Techniques"[mh] OR "Decision Trees"[mh] OR ((guideline*[tiab] OR ((decision[tiab] OR prediction*[tiab]) AND (making OR support*[tiab] OR tree*[tiab] OR model*[tiab] OR model*[tiab] OR algorithm*[tiab] OR triage*[tiab] OR protocol*[tiab] OR principle*[tiab] OR aid[tiab] OR aids[tiab]))) OR ((guideline*[tiab] OR decision*[tiab] OR model[tiab] OR tree[tiab] OR prediction*[tiab] OR model*[tiab] OR algorithm*[tiab] OR triage*[tiab] OR protocol*[tiab] OR principle*[tiab]) AND (develop*[tiab] OR propose*[tiab] OR new OR novel OR validat*[tiab] OR Evaluat*[tiab] OR implement*[tiab] OR modif*[tiab])))) AND (child[mh] OR infant[mh] OR "pediatrics"[mh] OR (preschool*[tiab] OR child*[tiab] OR schoolchild*[tiab] OR infan*[tiab] OR toddler*[tiab] OR pediatric*[tiab] OR paediatric*[tiab])) AND english[la] NOT (letter[pt] OR news[pt] OR comment[pt] OR editorial[pt] OR congresses[pt] OR abstracts[pt]) AND publisher[sb]

Google scholar First 200
Pneumonia guideline|"decision|prediction making|support|tree|model|model|aid|aids"
child|children|schoolchild|infants|pediatric|paediatric antibiotic|antibiotics|"anti biotic"
allintitle:Pneumonia guideline|"decision|prediction making|support|tree|model|model|aid|aids"
child|children|schoolchild|infants|pediatric|paediatric

Update literature search, September 2017

Database	Number of articles	Number of articles after deleting duplicates
Embase.com	3092	3041
Medline Ovid	1763	369
Web of science	1730	726
Google scholar	200	150
**Total**	6785	4286

In- and exclusion criteria for systematic review

Characteristic	Inclusion	Exclusion
**Design**	Studies assessing diagnostic accuracy or deriving or validating prediction models	Reviews, conference abstracts, letters, notes, editorials, news, comments
**Participants**	Children aged 1month to 5 years are substantial part (>50%) of the population.	Out of age range, children with severe comorbidity
**Setting**	Developed countries (based on the United Nations classification)<br>Emergency department	Developing countries<br>Primary care, in-hospital setting
**Intervention**	Multifactor clinical prediction rule including clinical features (and biomarkers).	Rules without clinical features, or including tests not available at ED
**Outcome**	(bacterial) pneumonia<br>treatment advice for pneumonia	Other diagnosis
