# Supplementary material for: Can clinical prediction models assess antibiotic need in childhood pneumonia? A validation study in paediatric emergency care
Source: PLoS One. 2019 Jun 13;14(6):e0217570. doi: 10.1371/journal.pone.0217570 (PMC6563975; doi:10.1371/journal.pone.0217570)
Supplement: S1 Fig — (PDF) [file pone.0217570.s002.pdf]

Supplementary File 1 Figure. Classification of febrile illness (based on Herberg [1])

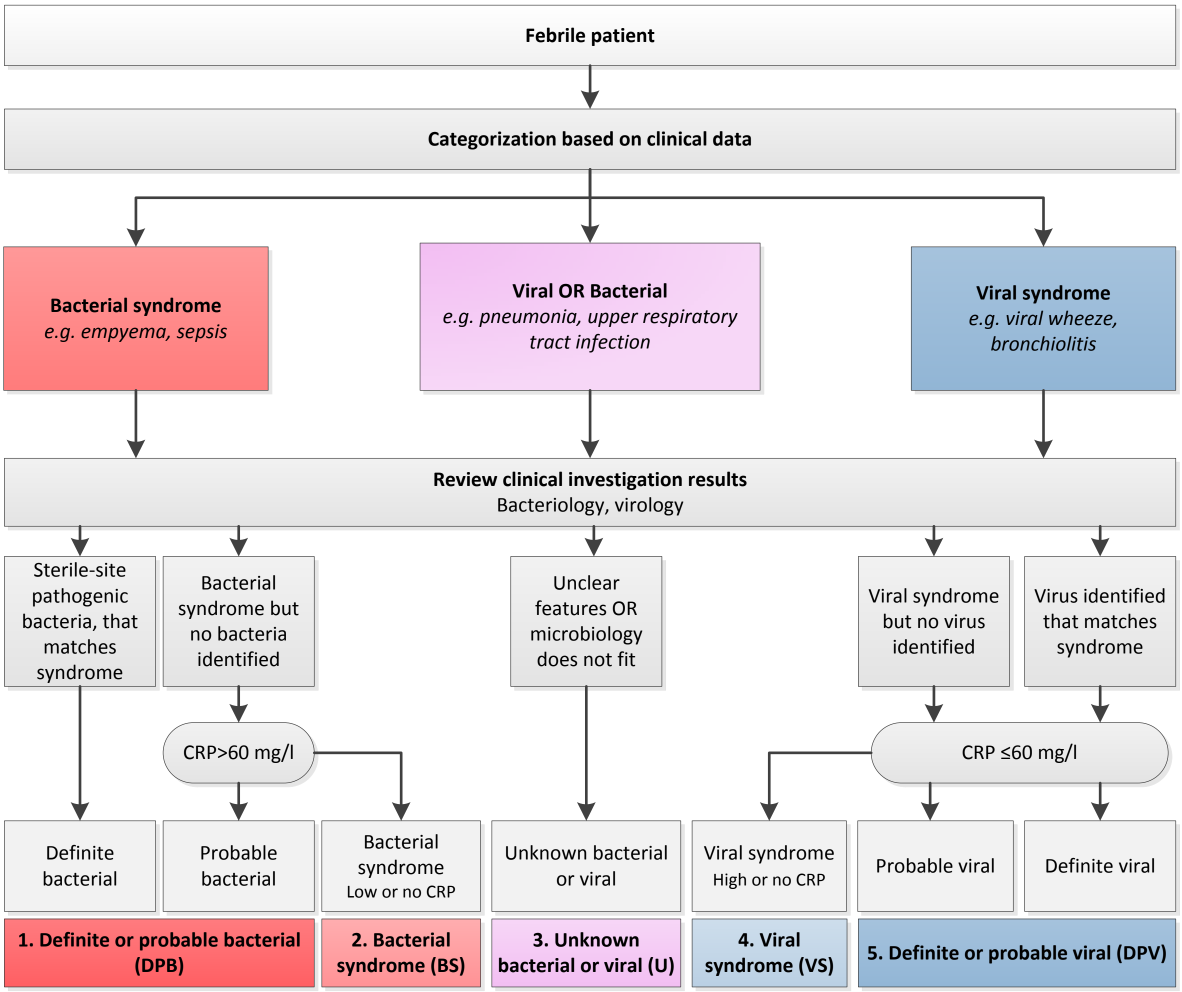

1. Herberg JA, Kaforou M, Wright VJ, Shailes H, Eleftherohorinou H, Hoggart CJ et al. Diagnostic Test Accuracy of a 2-Transcript Host RNA Signature for Discriminating Bacterial vs Viral Infection in Febrile Children. Jama. 2016;316(8):835-45.
