# Supplementary figures and images for: Can clinical prediction models assess antibiotic need in childhood pneumonia? A validation study in paediatric emergency care
Source: PLoS One. 2019 Jun 13;14(6):e0217570. doi: 10.1371/journal.pone.0217570 (PMC6563975; doi:10.1371/journal.pone.0217570)

Supplementary Material 2 Figure. Flowchart of the selection process

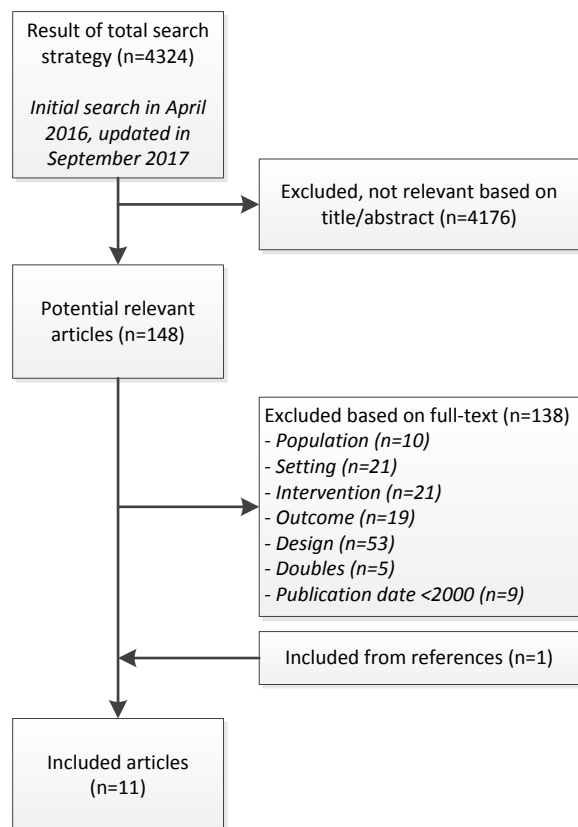

Supplement: S2 Fig — (PDF) [file pone.0217570.s003.pdf]

Supplementary Material 3 Figure. QUADAS-2 assessment

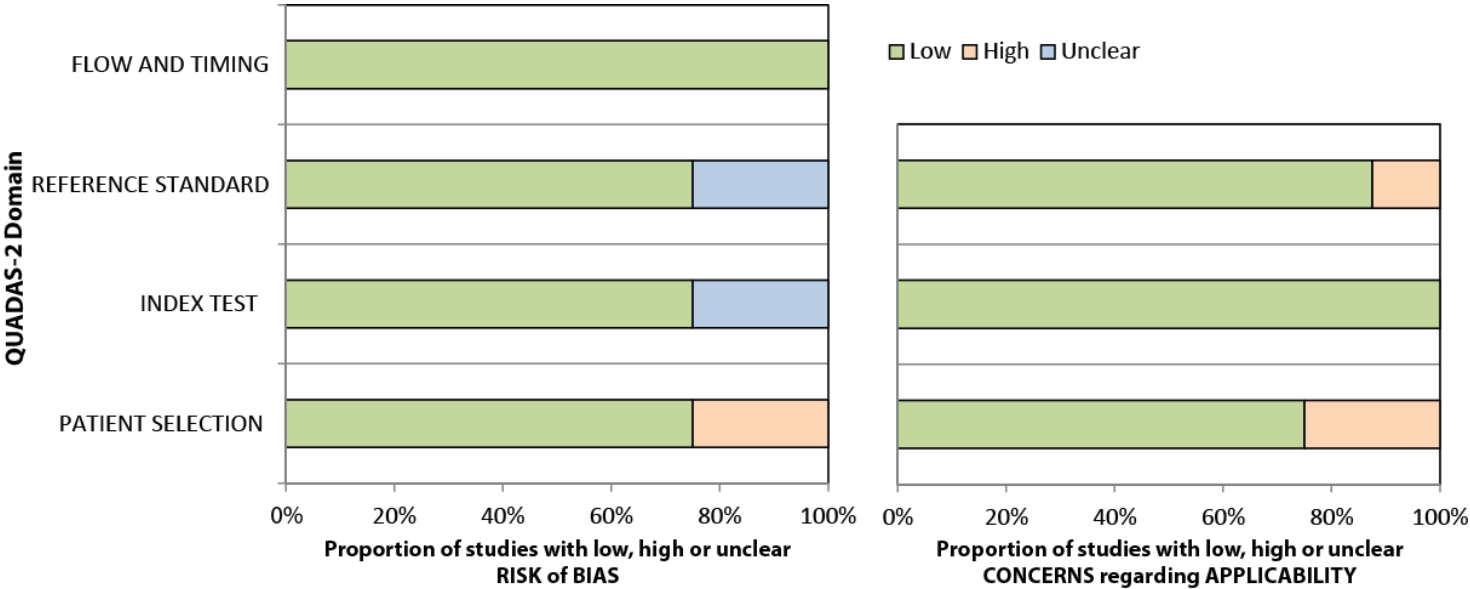

Supplement: S3 Fig — (PDF) [file pone.0217570.s004.pdf]
