# Supplementary material for: Can clinical prediction models assess antibiotic need in childhood pneumonia? A validation study in paediatric emergency care
Source: PLoS One. 2019 Jun 13;14(6):e0217570. doi: 10.1371/journal.pone.0217570 (PMC6563975; doi:10.1371/journal.pone.0217570)
Supplement: S4 Fig — (PDF) [file pone.0217570.s005.pdf]

Supplementary Material 4 Figure. Performance Model Lynch

Rotterdam

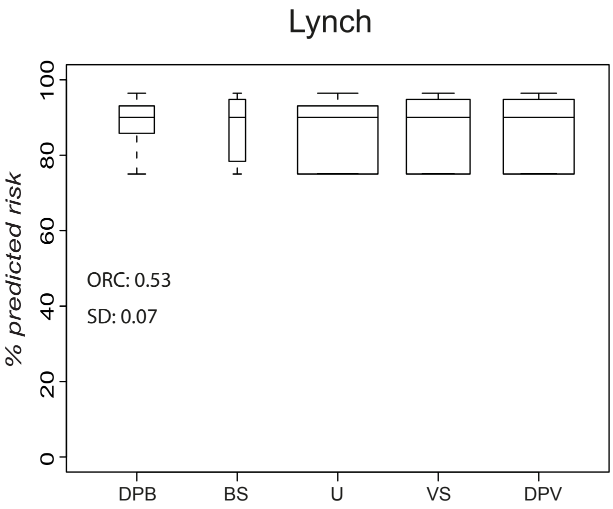

DPB = definite or probable bacterial, BS = bacterial syndrome, U = unknown, VS = viral syndrome, DPV = definite or probable viral; ORC = ordinal c-statistic; SD = standard deviation
