# Supplementary material for: Can clinical prediction models assess antibiotic need in childhood pneumonia? A validation study in paediatric emergency care
Source: PLoS One. 2019 Jun 13;14(6):e0217570. doi: 10.1371/journal.pone.0217570 (PMC6563975; doi:10.1371/journal.pone.0217570)
Supplement: S1 Table — (PDF) [file pone.0217570.s006.pdf]

Supplementary Material 1 Table. Missings and proxies of predictor variables

	Rotterdam, n=248		Coventry, n=301	
Predictor variables	*Missing, n (%)*	*Proxy*	*Missing, n (%)*	*Proxy*
Age, months	none		none	
Gender	none		none	
Temperature (°C)	12 (5)		4 (1.3)	
Duration of fever (days)	189 (76)		301 (100)	Derivation population
Respiratory rate	65 (26)		43 (14)	
Heart rate	41 (17)		7 (2)	
Oxygen saturation (%)	76 (30)		15 (5)	
Ill appearance	99 (40)		none	Toxic appearance
Dyspnea	none			
Decreased breath sounds	112 (45)		301 (100)	NA
Crackles	121 (49)		301 (100)	NA
Focal rales	97 (39)		301 (100)	NA
Retractions	143 (57)		301 (100)	NA
Nasal flaring	190 (77)		301 (100)	Dyspnea
Prolonged capillary refill (>2sec)	53 (21)		114 (38)	
CRP, mg/L	154 (62)		192 (64)	
Normal air entry		Absence of dyspnea		Absence of dyspnea
Resistin, ng/mL	248 (100)	Derivation population	301 (100)	Derivation population
PCT, ug/L	248 (100)	Derivation population	301 (100)	Derivation population

NA = not assessed, no close proxy available
