# Supplementary material for: Can clinical prediction models assess antibiotic need in childhood pneumonia? A validation study in paediatric emergency care
Source: PLoS One. 2019 Jun 13;14(6):e0217570. doi: 10.1371/journal.pone.0217570 (PMC6563975; doi:10.1371/journal.pone.0217570)
Supplement: S2 Table — (PDF) [file pone.0217570.s007.pdf]

Supplementary Material 2 Table. Detailed classification of risk groups based on different prediction models

Nijman Rotterdam

Antibiotic prescription			Clinical diagnosis					Total
			DPB	BS	U	VS	DPV	
No	Predicted risk	0 - 10 %	0	2	40	28	44	114
		10 - 15%	0	0	15	11	4	30
		>15 %	0	0	27	19	7	53
	Total		0	2	82	58	55	197
Yes	Predicted risk	0 - 10 %	3	2	3	0	8	16
		10 - 15%	3	0	2	0	2	7
		>15 %	12	0	7	1	8	28
	Total		18	2	12	1	18	51

**Threshold 10%**	n	**%**
Total number of children below threshold	130	**52%**
Number of treated children below threshold	16	**6%**
Number of treated children with bacterial infection below threshold	5	**2%**
Antibiotic prescription when guided by threshold 10%	35	14%
**Threshold 15%**		
Total number of children below threshold	167	**67%**
Number of treated children below threshold	23	**9%**
Number of treated children with bacterial infection below threshold	8	**3%**
Antibiotic prescription when guided by threshold 15%	28	11%

Nijman Coventry

Antibiotic prescription			Clinical diagnosis					Total
			DPB	BS	U	VS	DPV	
No	Predicted risk	0 - 10 %	4	4	67	41	21	137
		10 - 15%	1	0	15	6	1	23
		>15 %	3	0	20	11	2	36
	Total		8	4	102	58	24	196
Yes	Predicted risk	0 - 10 %	6	9	12	10	19	56
		10 - 15%	5	2	1	2	3	13
		>15 %	19	1	5	3	8	36
	Total		30	12	18	15	30	105

**Threshold 10%**		
Total number of children below threshold	193	**64%**
Number of treated children below threshold	56	**19%**
Number of treated children with bacterial infection below threshold	15	**5%**
Antibiotic prescription when guided by threshold 10%	49	16%
**Threshold 15%**		
Total number of children below threshold	229	**76%**
Number of treated children below threshold	69	**23%**
Number of treated children with bacterial infection below threshold	22	**7%**
Antibiotic prescription when guided by threshold 15%	36	12%

Oostenbrink Rotterdam

Antibiotic prescription			Clinical diagnosis					Total
			DPB	BS	U	VS	DPV	
No	Predicted risk	0 - 10 %	0	2	17	16	27	62
		10 - 15%	0	0	6	12	14	32
		>15 %	0	0	59	30	14	103
	Total		0	2	82	58	55	197
Yes	Predicted risk	0 - 10 %	0	0	1	0	6	7
		10 - 15%	2	0	1	0	6	9
		>15 %	16	2	10	1	6	35
	Total		18	2	12	1	18	51

**Threshold 10%**		
Total number of children below threshold	69	**28%**
Number of treated children below threshold	7	**3%**
Number of treated children with bacterial infection below threshold	0	**0%**
Antibiotic prescription when guided by threshold 10%	44	18%
**Threshold 15%**		
Total number of children below threshold	110	**44%**
Number of treated children below threshold	16	**6%**
Number of treated children with bacterial infection below threshold	2	**1%**
Antibiotic prescription when guided by threshold 15%	35	14%

Oostenbrink Coventry

Antibiotic prescription			Clinical diagnosis					Total
			DPB	BS	U	VS	DPV	
No	Predicted risk	0 - 10 %	1	2	33	22	8	66
		10 - 15%	1	2	24	19	12	58
		>15 %	5	0	46	17	4	72
	Total		7	4	103	58	24	196
Yes	Predicted risk	0 - 10 %	3	5	6	4	10	28
		10 - 15%	1	4	5	2	14	26
		>15 %	26	3	8	8	6	51
	Total		30	12	19	14	30	105

**Threshold 10%**		
Total number of children below threshold	94	**31%**
Number of treated children below threshold	28	**9%**
Number of treated children with bacterial infection below threshold	8	**3%**
Antibiotic prescription when guided by threshold 10%	77	26%
**Threshold 15%**		
Total number of children below threshold	178	**59%**
Number of treated children below threshold	54	**18%**
Number of treated children with bacterial infection below threshold	13	**4%**
Antibiotic prescription when guided by threshold 15%	51	17%

Irwin Rotterdam

Antibiotic prescription			Clinical diagnosis					Total
			DPB	BS	U	VS	DPV	
No	Predicted risk	0 - 10 %	0	1	29	19	38	87
		10 - 15%	0	0	11	4	0	15
		>15 %	0	1	42	35	17	95
	Total			2	82	58	56	197
Yes	Predicted risk	0 - 10 %	4	1	5	1	2	13
		10 - 15%	3	0	2	0	0	5
		>15 %	11	1	5	0	16	33
	Total		18	2	12	1	18	51

		%
**Threshold 10%**		
Total number of children below threshold	100	**40%**
Number of treated children below threshold	13	**5%**
Number of treated children with bacterial infection below threshold	5	**2%**
Antibiotic prescription when guided by threshold 10%	38	15%
**Threshold 15%**		
Total number of children below threshold	120	**48%**
Number of treated children below threshold	18	**7%**
Number of treated children with bacterial infection below threshold	8	**3%**
Antibiotic prescription when guided by threshold 15%	33	13%

Irwin Coventry

Antibiotic prescription			Clinical diagnosis					Total
			DPB	BS	U	VS	DPV	
No	Predicted risk	0 - 10 %	3	3	58	32	18	114
		10 - 15%	2	0	19	5	1	27
		>15 %	2	1	26	21	5	55
	Total		7	4	103	58	24	196
Yes	Predicted risk	0 - 10 %	7	8	10	3	13	41
		10 - 15%	6	1	3	5	1	16
		>15 %	17	3	6	6	16	48
	Total		30	12	19	14	30	105

**Threshold 10%**		
Total number of children below threshold	155	**51%**
Number of treated children below threshold	41	**14%**
Number of treated children with bacterial infection below threshold	15	**5%**
Antibiotic prescription when guided by threshold 10%	64	21%
**Threshold 15%**		
Total number of children below threshold	198	**66%**
Number of treated children below threshold	57	**19%**
Number of treated children with bacterial infection below threshold	22	**7%**
Antibiotic prescription when guided by threshold 15%	48	16%
